# Divergent and convergent creativity relate to different aspects of semantic control

**DOI:** 10.1162/imag_a_00502

**Published:** 2025-03-07

**Authors:** Katya Krieger-Redwood, Lucilla Lanzoni, Tirso R.J. Gonzalez Alam, Rebecca L. Jackson, Jonathan Smallwood, Elizabeth Jefferies

**Affiliations:** Department of Psychology, York Neuroimaging Centre, York Biomedical Research Institute, University of York, York, United Kingdom; School of Psychology and Sports Science, Bangor University, Bangor, United Kingdom; Department of Psychology, Queen’s University, Kingston, Ontario, Canada

**Keywords:** creativity, semantic control, connectivity, ATL, IFG, functional architecture

## Abstract

Past work has demonstrated a link between semantic memory and verbal creativity. Yet, few studies have considered this relationship through the lens of the controlled semantic cognition account, which anticipates that multimodal concepts in long-term memory interact with semantic control processes to generate goal and context-appropriate patterns of retrieval. In particular, while the creativity literature has distinguished divergent and convergent aspects of creativity, little is known about their relationship with separable aspects of semantic control, or the semantic intrinsic functional architecture of the brain. We investigated whether tasks with greater reliance on controlled semantic retrieval (assessed through weak association) versus semantic selection (assessed through semantic feature matching) were differentially linked to divergent creativity (assessed with the unusual uses task; UUT) and convergent creativity (assessed with the remote associates task; RAT). Better performance on the RAT was linked to semantic selection, while stronger performance on UUT was linked to more efficient retrieval of weak associations. We also examined individual differences in the intrinsic functional architecture of the semantic system using resting-state fMRI. Greater coupling between the anterior temporal lobe (multimodal semantic store) and left inferior frontal gyrus (LIFG) (in the semantic control network) was linked to stronger convergent creativity. This pathway also correlated with semantic feature matching performance, but not the retrieval of weak associations. In contrast, better divergent creativity was linked to greater coupling between LIFG and language-related auditory-motor regions, and decoupling from the default mode and frontoparietal networks. These connections correlated with the retrieval of weak associations. Interestingly, while*decoupling*of LIFG with default mode and frontoparietal networks correlated with the retrieval of weak associations,*coupling*of LIFG with these networks correlated with semantic feature matching. These behavioural and neurocognitive dissociations show that semantic control and creativity are highly related yet multifaceted constructs that depend on the underlying intrinsic architecture of key sites related to semantic cognition.

## 1 Introduction

Semantic cognition pervades all aspects of life, making it necessary to flexibly deploy concepts to suit the current context or goal. Semantic cognition involves at least two components: representation of long-term knowledge, which is thought to depend on multimodal conceptual representations within the anterior temporal lobe (Binder et al., 2009;Patterson et al., 2007;Rogers et al., 2006;Snowden et al., 2001,^2004^;Visser et al., 2009,^2010^;Warrington, 1975), and control processes that act upon this conceptual “store” supported by a left-lateralised semantic control network (Gao et al., 2021;Jackson, 2021;Wang et al., 2020). For example, presented with the words “dog” and “bone”, people can quickly identify the link between the two words, with little need to exert control over retrieval. However, the words “dog” and “bookcase,” likely necessitate the engagement of other processes to generate a viable link (e.g., a bookcase containing books about training dogs).

Semantic concepts often need to be deployed creatively when solving verbal problems. Research has distinguished two aspects of verbal creativity (Green et al., 2023). Divergent creativity tasks generally involve generating multiple uses for a single stimulus, for example, given the word “brick,” unusual uses for this item must be produced. In contrast, convergent creativity tasks generally involve generating a single response that draws the presented stimuli together, for example, generating the word “cheese” to link “Swiss,” “cottage,” and “cake.” While these tasks share some processes, divergent creativity involves generating multiple solutions to a problem, through exhaustive memory search, and convergent creativity relies on an interplay of control and memory to expand the search space while bypassing related concepts that are irrelevant to the current goal (Beaty & Kenett, 2023). Children’s convergent creative ability develops before divergent creativity—which follows on around the age of 12 years—this catch up in divergent thinking is thought to be due to an increase in abstract thinking around this age (Eon Duval et al., 2023), and suggests that the two types of creativity leverage somewhat different processes. Furthermore, although successful completion of convergent and divergent tasks relies on the default mode (generating ideas via associative thinking/memory), salience (identifying promising candidate ideas), and executive (idea evaluation, selection, modification) networks (Beaty et al., 2014,^2015^;Beaty & Kenett, 2023;Ovando-Tellez, Benedek, et al., 2022;Zhang et al., 2023), meta-analytic results suggest their activation profiles do also diverge (Gonen-Yaacovi et al., 2013). Performance on verbal creativity tasks has also been linked to semantic memory structure: studies have shown that participants with a more well-connected and flexible memory structure perform better on divergent and convergent creativity tasks, as well as metrics of real-life creativity (Beaty & Kenett, 2023;Benedek et al., 2017;Bernard et al., 2019;He et al., 2021;Kenett et al., 2014;Kenett & Faust, 2019;Luchini et al., 2023;Ovando-Tellez, Kenett, et al., 2022).

While studies have examined the link between semantic memory and verbal creativity, few studies have considered this relationship through the lens of the Controlled Semantic Cognition account, which anticipates that concepts interact with semantic control processes supported by a left-lateralised semantic control network (SCN) of regions in frontal and posterior temporal cortex (Lambon Ralph et al., 2017). In a recent study, we demonstrated that the SCN responds both when participants process weak associations and when they generate more unusual or creative responses (Krieger-Redwood et al., 2022), suggesting that semantic control processes are important in verbal creativity. Another recent study byBenedek et al. (2020)required participants to generate associations based on the visual features of the stimulus (e.g., red-round = “clown’s nose”), and activation fell outside of control networks, in angular and lingual gyri, suggesting a visually mediated strategy for generating these associations; however, more original associations also activated control regions such as LIFG and SMA. These two studies alone highlight how concepts can be manipulated and deployed in*different*ways in service of a task requiring verbal creativity.

These studies demonstrate an overlap in neurocognitive processing for creativity and semantic control, but they do not consider whether different aspects of semantic control are leveraged depending on the creative process engaged.Ovando-Tellez, Kenett, et al. (2022)designed an associative fluency task (AFT) using polysemous words (for example, bank—meaning riverbank or financial institution), and, using principal components analysis, revealed that participants generated responses in two different ways: responses either (i) clustered*within*a semantic meaning or (ii) switched*between*meanings. These two components correlated with aspects of creativity: (i) for divergent thinking (i.e., the alternate uses task), fluency and number of unique ideas generated correlated with the component capturing AFT responses clustering within a semantic meaning; while (ii) convergent thinking (measured using the “combination of associates” task) correlated with the ability to switch between polysemous meanings. This suggests that components of creativity relate in different ways to aspects of semantic cognition.

At the same time, research on semantic control suggests that partially distinct processes underpin the controlled retrieval of weak associations versus the selection of specific features of concepts in line with a goal (Badre et al., 2005;Wang et al., 2024;Whitney et al., 2012). Association matching tasks manipulate the semantic distance between words—for weakly associated words (e.g., “letter” and “quill”), the relationship between the items is less strong, and participants take longer to arrive at a decision for weak than strong association trials. Feature selection requires matching items based on a specific feature of a concept, rather than the concept as a whole, for example, pairing “post-box” and “tomato” because they share the same colour. These tasks often also include the requirement to inhibit a pre-potent distractor, for example, rejecting “letter” in favour of “tomato” as a match for “post-box”. Semantic control tasks tend to produce activation across brain regions implicated in domain-specific (i.e., semantic) and domain-general control, and the two partially overlap (Fedorenko et al., 2013;Jackson, 2021).

However, there is some evidence to suggest that both shared and separable aspects of semantic control might be engaged across different types of semantic decision. For example, classic studies, such as those fromBadre and Wagner (2007),Badre et al. (2005), andThompson-Schill et al. (1997), demonstrated that while activation overlapped across middle portions of LIFG, the response diverged anterior/ventrally for associative judgements and dorsally for feature selection. This suggests that the two types of semantic decision engage both overlapping*and*distinct control processes. This was also seen in a recent study which used a yes/no decision paradigm (rather than 3 or 4 AFC): although activation was similar for the weak association and feature selection, activation spread anteriorly for association judgements and towards domain-general regions for feature matching (Wang et al., 2024). Furthermore,Chiou et al. (2018)recently demonstrated that connectivity between semantic control, representation, and sensorimotor regions changes depending on whether the semantic decision is based on association or features of a concept. Therefore, given that exerting control over semantic memory recruits both shared and divergent processes dependent on the context (or task/goal at hand;January et al., 2009;Kan et al., 2003;Moss et al., 2005;Novick et al., 2010;Thompson-Schill et al., 2005,^2009^), and the literature demonstrating clear links between verbal creativity and semantic memory—it could be the case that different aspects of semantic control link to different aspects of verbal creativity.

Therefore, this study sought to uncover the relationship between these differentiable semantic control processes and divergent and convergent creativity. We used two well-established semantic control tasks, requiring participants to select a target based on either a weak global association or a specific feature relationship, and compared these tasks with the retrieval of strong global associations (e.g., letter–envelope), which places low demands on semantic control. We also employed two widely used verbal creativity tasks, the unusual uses task (UUT) to measure divergent creativity, and the remote associates task (RAT) to capture convergent creativity. Our predictions follow on from the work ofOvando-Tellez, Kenett, et al. (2022), who found that divergent thinking correlates with broad retrieval ability for semantic concepts: UUT requires participants to identify meaningful contexts in which an object could be used, and weak associations also require the generation of linking contexts. In contrast, the RAT requires selective retrieval of information that is highly constrained by the words provided in each trial and, therefore, may overlap with semantic control processes required for semantic feature selection tasks. In line with this prediction,Ovando-Tellez, Kenett, et al. (2022)found that performance on a convergent creativity task was linked to switching between meanings, allowing efficient selection of conceptual information. In addition, we obtained a resting-state scan, allowing us to uncover how the intrinsic neural architecture underpinning semantic cognition relates to performance on divergent and convergent creativity tasks. We examined two seeds relevant to semantic cognition: left anterior temporal lobe, a region implicated in long-term semantic representation (e.g.,Balgova et al., 2022;Binney et al., 2010,^2016^;Chen et al., 2016;Jefferies, 2013;Lambon Ralph et al., 2009,^2010^,^2012^,^2015^;Malone et al., 2016;Pobric et al., 2007), and LIFG, implicated across a wide range of semantic control tasks in a recent meta-analysis of semantic control (Jackson, 2021). While previous research indicates that there may be some differences along LIFG in its recruitment for semantic feature selection versus weak association, the peak LIFG SCN site should be relevant to both tasks, given that (i) it is derived from a large-scale meta-analysis of semantic control, (ii) the finding fromBadre and Wagner (2007)andBadre et al. (2005)association and selection tasks overlap in mid-LIFG, and (iii) recent studies suggesting that connectivity from LIFG changes based on the demands of the task (Chiou et al., 2018;Gao et al., 2022).

## 2 Method

Participants underwent laboratory-based behavioural testing sessions, 2 hours in duration, on 2 consecutive days. They performed a large battery of computer-based tasks, including the semantic association tasks, unusual uses task (UUT), remote associates task (RAT), and a shortened version (18 questions) of the Raven’s Advanced Progressive Matrices (RAPM), as well as many measures unrelated to this study, such as those related to mind-wandering, executive control, and episodic memory (Evans et al., 2020;Karapanagiotidis et al., 2017;Poerio et al., 2017;Sormaz et al., 2017,^2018^;Turnbull et al., 2019;Vatansever et al., 2017;H.-T. Wang et al., 2017,^2018^;Wang et al., 2018).

Our sample size was constrained by several characteristics, detailed below (Section 2.1), and we report all data exclusions, all inclusion/exclusion criteria, whether inclusion/exclusion criteria were established prior to data analysis, all manipulations, and whether participants completed all measures of interest for the current study. This study was not pre-registered in a time-stamped, institutional registry prior to the research being conducted.

### 2.1 Participants

All participants were right-handed, native English speakers with normal/corrected vision, and compensated for their time with payment or course credit. We analysed data from a large cohort of 165 volunteers recruited from the University of York (99 females, mean age = 20.3, range = 18–31 years) who completed a resting-state scan, followed by cognitive tests in subsequent sessions on different days (for a full list of tasks, seeSupplementary Materials: “Battery of Tasks”). These data have been used in previous studies focused on the lateralisation of semantic cognition (Gonzalez Alam et al., 2019), cortical thickness (Wang et al., 2018), neurocognitive components of semantic performance (Vatansever et al., 2017), mind-wandering (Poerio et al., 2017;Sormaz et al., 2018;Turnbull et al., 2019;H.-T. Wang et al., 2017,^2018^), and hippocampal connectivity (Karapanagiotidis et al., 2017;Sormaz et al., 2017). We excluded 50 participants: 31 due to missing behavioural data (i.e., they did not complete all of the tasks used in this study), 11 due to low accuracy (<40%) on the semantic tasks, 2 extreme behavioural outliers (3xIQR for efficiency (RT/accuracy; see last paragraph ofSection 2.4.2for more details) in the weak association task), 1 due to missing MRI data, 1 due to the use of an incorrect TR during MRI acquisition, and 4 during pre-processing because they exceeded our motion cut-off of 0.3 mm average displacement, had more than 20% invalid scans (identified as outliers during outlier detection based on GS and framewise displacement), and/or a mean global signal change of z > 2. The final sample size, therefore, consisted of 115 participants (71 females, mean age = 20, range = 18–31 years). None of the participants had a history of psychiatric or neurological illness, drug use that could alter cognitive functioning, severe claustrophobia, or pregnancy. All volunteers provided written informed consent and were debriefed after data collection. Ethical approval was obtained from Ethics Committees in the Department of Psychology and York Neuroimaging Centre, University of York.

### 2.2 MRI data acquisition

Structural and functional MRI data were acquired using a 3T GE HDx Excite MRI scanner utilising an eight-channel phased array head coil tuned to 127.4 MHz, at the York Neuroimaging Centre, University of York. Structural MRI acquisition was based on a T1-weighted 3D fast spoiled gradient echo sequence (TR = 7.8 seconds, TE = minimum full, flip angle = 20°, matrix size = 256 x 256, 176 slices, voxel size = 1.13 x 1.13 x 1 mm^3^). Resting-state fMRI data were recorded from the whole brain using single-shot 2D gradient-echo-planar imaging (TR = 3 seconds, TE = minimum full, flip angle = 90°, matrix size = 64 x 64, 60 slices, voxel size = 3 x 3 x 3 mm^3^, 180 volumes). Participants passively viewed a fixation cross and were not asked to think of anything in particular for the duration of the scan (9 minutes). A T1-weighted FLAIR scan with the same orientation as the functional scans was collected to improve co-registration between subject-specific structural and functional scans (TR = 2560 ms, TE = minimum full, matrix size = 64 x 64, voxel size = 3 x 3 x 3 mm^3^).

### 2.3 Pre-processing

Pre-processing was performed using the CONN functional connectivity toolbox V.20b (http://www.nitrc.org/projects/conn;Whitfield-Gabrieli & Nieto-Castanon, 2012). Functional volumes were slice-time (bottom-up, interleaved) and motion-corrected, skull-stripped, and co-registered to the high-resolution structural image, spatially normalised to Montreal Neurological Institute (MNI) space using the unified-segmentation algorithm, smoothed with a 6 mm FWHM Gaussian kernel, and band-passed filtered (0.008–0.09 Hz) to reduce low-frequency drift and noise effects. A pre-processing pipeline of nuisance regression included motion (12 parameters: the 6 translation and rotation parameters and their temporal derivatives), scrubbing (all outlier volumes were identified through the artefact detection algorithm included in CONN, with conservative settings: scans for each participant were flagged as outliers based on scan-by-scan change in global signal above z = 3, subject motion threshold above 5 mm, differential motion and composite motion exceeding 95% percentile in the normative sample), and CompCor components (the first five) attributable to the signal from white matter and CSF (Behzadi et al., 2007), as well as a linear detrending term, eliminating the need for global signal normalisation (Chai et al., 2012;Murphy et al., 2009).

### 2.4 Tasks

#### 2.4.1 Semantic association task

This task employed a three-alternative forced-choice design: participants matched a coloured probe picture with one of three possible target words, pressing one of three buttons to indicate the word that was most strongly associated with the probe picture. We manipulated strength of association between the probe and target, resulting in strong association (low control) and weak association (high control) trials. The trials were created using associations derived from free association databases (e.g., Edinburgh Association Thesaurus). Strength of association was assessed using ratings on a 7-point scale (from a different set of participants) and differed significantly between conditions (Supplementary Table S1). As an additional confirmation of the distance between probe and target, we also computed word2vec scores for the probe–target relationships. Word2vec (Mikolov et al., 2013) uses word co-occurrence patterns in a large language corpus to derive semantic features for items, which can then be compared to determine their similarity. The word2vec score for the probe–target relationship differed significantly (*t*(26) = 4.24,*p*< .001) between the high (mean w2v = .3, SD = .15) and low (mean w2v = .2, SD = .11) conditions. The pictures and words were also rated for familiarity using a 7-point scale (from a different set of participants), and lexical frequency for the words was obtained from the SUBTLEX-UK database (van Heuven et al., 2014). Additional psycholinguistic data were taken from the MRC psycholinguistic database (Coltheart, 1981;Wilson, 1988): there were no differences between strong and weak associations in familiarity, word length, lexical frequency, or imageability (Supplementary Table S1).

The stimuli were selected from a larger set of words and photographs used in previous experiments (Davey et al., 2015;Krieger-Redwood et al., 2015). The pictures were photographs sourced from the internet and re-sized (200 pixels, 72 dpi). The distractors were unrelated to the probe and were targets on other trials. We presented 60 coloured pictures of objects (e.g., dog), paired with 60 strongly related (e.g., bone) and 60 weakly related (e.g., ball) written words, resulting in 120 trials. These trials were presented in 4 blocks of 30 trials each, with both conditions interspersed in each block. The order of trials within the blocks was randomised across subjects. The blocks were interleaved with other types of semantic judgements and non-semantic judgements outside the scope of this report.

**Fig. 1.**
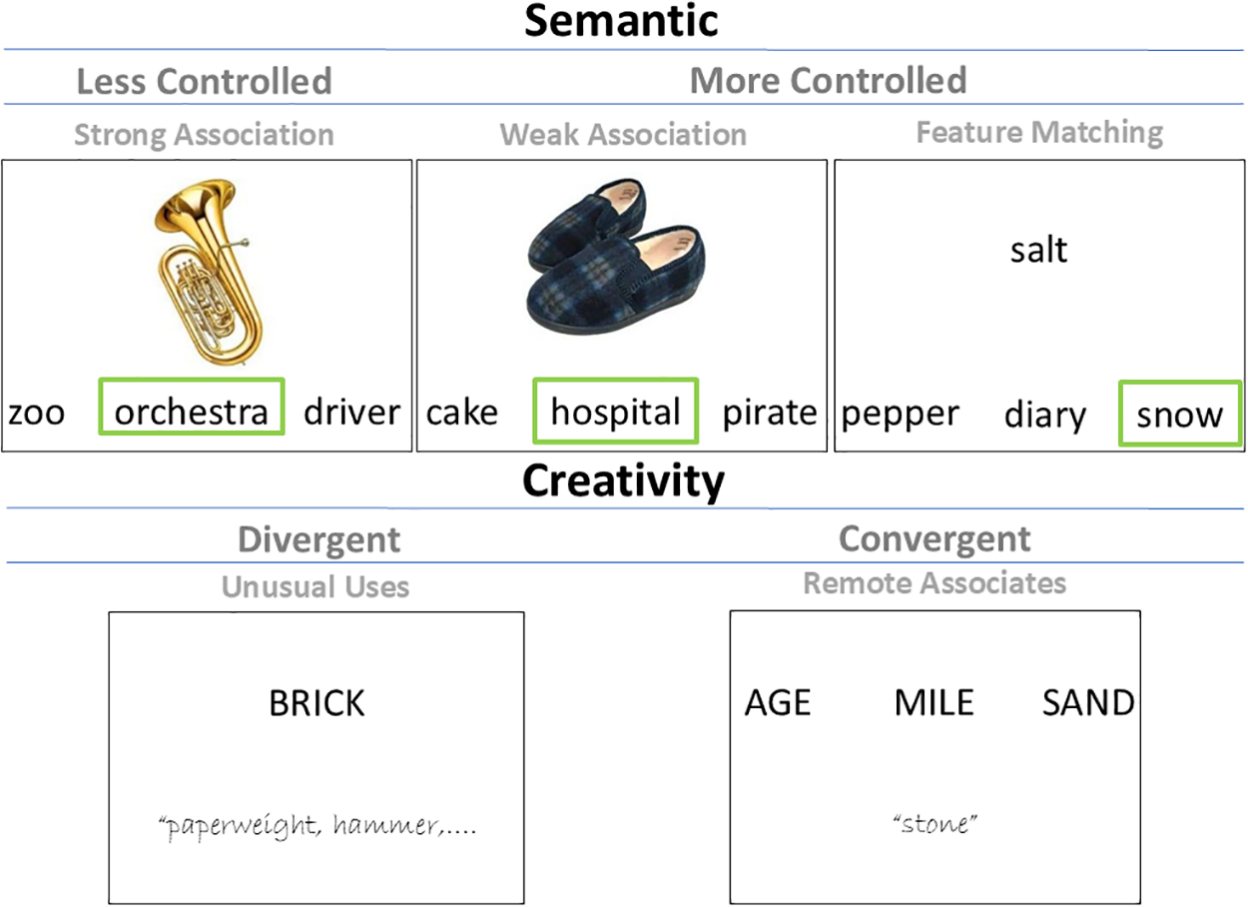
Task conditions. Top: The two tasks requiring a high degree of semantic control involved weak association matching and feature matching. These tasks were compared with strong association matching which requires less semantic control. Bottom: The creativity tasks. The unusual uses task required participants to generate as many items as possible for an object in 2 minutes, tapping into divergent thinking. The remote associates tasks required participants to generate a response linking the three concepts, tapping into convergent thinking.

Each trial started with a blank screen for 500 ms. The response options were subsequently presented at the bottom of the screen for 900 ms (with the three options aligned horizontally, and the target in each location equally often). Finally, the probe was centrally presented at the top of the screen. The probe and choices remained visible until the participant responded, or for a maximum of 3 seconds (Fig. 1;Supplementary Fig. S1summarises behavioural results).

#### 2.4.2 Semantic feature matching task

In the feature matching task (adapted fromBadre et al., 2005), participants had to select the target that matched a probe according to a semantic feature (colour, size, shape, or texture). A strong semantic associate was also presented among the choices (e.g., colour–salt:***dove***, corn,*pepper*), such that target retrieval required the explicit selection of the appropriate semantic feature (e.g., white) and the suppression of the dominant but irrelevant global associate (e.g., pepper). To capture the distance between probe and target in the feature matching task, we calculated the word2vec score between the probe and the lure (word2vec = .34). Furthermore, there was a significant difference in probe–distractor relationships between the weak association and feature selection tasks (*t*(57) = 4.03,*p*< .001), likely due to a greater spread in probe–distractor relationships in the feature selection (-.06 to .69) than the weak semantic association task (-.02 to .46). Thus, the feature matching task and the “weak” condition in the semantic association task (above) tapped two different aspects of semantic control (goal-driven selection of task-relevant semantic information and stimulus-driven controlled retrieval, respectively). Participants were asked to match based on the four different features in separate blocks, with instructions about the criterion to use for the matching at the beginning of each block and with a reminder present in each trial.

Each trial started with a blank screen for 500 ms. The probe and cue (in parentheses; colour, size, shape, or texture) were subsequently presented at the top of the screen for 1000 ms. Finally, the three answer choices were presented at the bottom of the screen (with the three options aligned horizontally, and the target in each location equally often). The probe, cue, and choices remained visible until the participant responded, or for a maximum of 3 seconds (Fig. 1;Supplementary Fig. S1summarises behavioural results). The target words were statistically comparable with those in the semantic associates task on mean word length and lexical frequency (seeSupplementary Table S2).

To capture possible speed–accuracy trade-offs, response efficiency (reaction time divided by accuracy) was calculated for all three semantic task conditions for each participant. Response efficiency scores were used in all behavioural and resting-state analyses.

#### 2.4.3 Unusual uses task

The unusual uses task (UUT;Guilford, 1967) is proposed to assess divergent thinking, an aspect of creativity. Three familiar objects were selected (newspaper, brick, and shoe); for each, the name appeared on screen for 10 seconds, followed by a response period of 2 minutes during which participants were required to type as many uses as they could (Fig. 1). Task instructions were as follows: “In this task you will be presented with the name of an object for 10 seconds. /You will then be taken to a blank screen where you are required to list as many uses for that object as you can think of in 2 minutes.” The response screen included the following text at the top of the screen; “Please list as many possible uses for that object as you can think of in 2 minutes. /Separate each use with comma”. Participants were not given explicit instructions to be creative when generating uses for the items. Therefore, the UUT scores in our study are a measure of incongruent divergent thinking (i.e., participants are scored on the creativity of their ideas, without having been explicitly told to be creative). This methodology may have limited the degree to which participants behaved creatively, as a recent meta-analysis suggests that the “be creative” instruction increases creativity and originality, but has no effect on fluency (Wei et al., 2024). However, it has also been argued that creative individuals develop a habit for uniqueness: even without explicit instruction to be creative, more unique ideas are produced by these individuals (Reiter-Palmon et al., 2019).

Word2vec (see above) was used as a method of scoring the uniqueness of the responses, in line with other studies using algorithm-based semantic distance scoring of UUT responses (Beaty & Johnson, 2021;Buczak et al., 2023;Dumas et al., 2021;Orwig et al., 2021). Our scoring differed slightly to these previous studies, with a simple two-step approach: (1) a human rater condensed each response into a single word to capture the response of the participant and (2) the distance between the probe (e.g., brick, shoe, or newspaper) and the response was measured using word2vec. Therefore, each use generated for the given object received a word2vec score, allowing us to quantify the distance between the object and the association made. For example, for the object “brick,” the response “fire” received a word2vec score of .148, due to its low association with “brick,” whereas “building” received .5, as this response is highly associated with the probe. Each participant’s word2vec scores were averaged across items and responses to create a single divergent thinking score, and reverse-scored so that better performance relates to a higher score like the other measures in this study. The final word2vec score correlated with human-rater scoring of the number of original responses (*r*= .54,*p*< .001), but not the total number of responses (i.e., fluency;*r*= .133,*p*= .16), suggesting that our word2vec scoring method was able to capture originality while removing the confound of fluency (Supplementary Table S3).

#### 2.4.4 Remote associates task

The remote associates task (RAT;Mednick, 1968) is a widely used measure of convergent aspects of creativity. Participants were presented with three cue words (e.g., cottage, swiss, cake) which link together by a fourth word (e.g., cheese). Participants were instructed to read the triads and to identify the linking word (Fig. 1). Triads were presented on screen for 5 seconds, then the triads disappeared and participants were instructed to type their answer. They were then asked whether they used insight to find the solution and given 5 seconds to respond yes or no. In total, 30 triads were used: these varied in difficulty (easy, medium, and hard trials) to maximise sensitivity to individual differences. Since in some trials, participants did not produce the target word but another semantically similar item, we used word2vec as our accuracy measure: for each trial, word2vec scores were calculated between the target and the response given by the participant. A higher word2vec score indicates a close response to the target, whereas a lower word2vec score indicates that the response was further away from the target. A similar scoring method was recently proposed byBeisemann et al. (2020), who found the construct validity was comparable with standard scoring. The correlation between binary accuracy and word2vec was very high (*r*(115) = .98,*p*< .001) and the brain–behaviour relationship was also consistent across both scoring methods (Supplementary Fig. S2). For reference, a table of normative data taken fromBowden and Jung-Beeman (2003)for each trial in the RAT and our word2vec accuracy data for each trial can be found in the Supplementary Materials (Supplementary Table S4).

### 2.5 Resting-state fMRI analysis

To establish an association between creativity and the neural architecture of semantic cognition, this analysis explored associations between performance on convergent and divergent creativity tasks and the intrinsic functional connectivity of spheres seeded in semantic representation and control regions. We placed spheres (radius = 6 mm) around peaks derived from meta-analyses for semantic representation (vATL (-41, -15, -31); coordinates averaged from table 1 “general semantics”;Rice, Hoffman, et al., 2018) and control (LIFG (-48, 22, 20);Jackson, 2021). There were two functional connectivity seed-to-voxel analyses; one for each seed (vATL, LIFG), assessing functional connectivity between the seed and every voxel in the brain. In a first-level analysis, we computed whole-brain seed-to-voxel correlations for each of our seeds. For the second-level analysis, we entered the z-scored average word2vec score for each participant for the remote associates task (RAT) and the unusual uses task (UUT) into a GLM analysis as explanatory variables (EVs), as well as a nuisance regressor corresponding to the mean motion for each participant (measured in framewise displacement). In all analyses, we convolved the signal with a canonical haemodynamic response function. We used two-sided tests to determine significant clusters. Group-level analyses in CONN were cluster-level FWE corrected and controlled for the number of seeds (Bonferroni,*p*< .025), and used a height threshold of*t*> 2.6). We used fslmaths to multiply our effects by the 7 networks established byYeo et al. (2011), and fslstats to count the voxels in these overlapping results.Supplementary Figure S3shows how much each of our effects overlaps with the seven resting-state networks fromYeo et al. (2011). An additional seed-to-voxel connectivity analysis can be found in Supplementary Materials, this analysis includes three ROI’s: posterior middle temporal gyrus (pMTG), a site implicated in semantic control (Jackson, 2021); and two LIFG sites identified byBadre et al. (2005)as important for either controlled selection or retrieval of semantic information (Supplementary Fig. S5).

The connectivity maps resulting from these analyses were uploaded to Neurovault (https://neurovault.org/collections/RUIVEQNY/;Gorgolewski et al., 2015). The conditions of our ethics approval do not permit public archiving of the raw MRI data supporting this study. Readers seeking access to these data should contact the lead author, Katya Krieger-Redwood, the PI Professor Beth Jefferies, or the local ethics committee at the Department of Psychology and York Neuroimaging Centre, University of York. Access will be granted to named individuals in accordance with ethical procedures governing the reuse of sensitive data. Specifically, the following conditions must be met to obtain access to the data: approval by the Department of Psychology and York Neuroimaging Research Ethics Committees and a suitable legal basis for the release of the data under GDPR. Scripts used for running the tasks used in this study were uploaded to OSF (https://osf.io/dy37q/?view_only=114b10e2fedc426f841cc615f7e6931d).

## 3 Results

### 3.1 Behavioural results

We examined whether there was any relationship between tasks that require semantic control and tasks that engage convergent and divergent creativity. We used a multivariate linear regression (SPSS; version 26) in which performance on semantic tasks (efficiency scores for feature matching, weak and strong association) was explanatory variables and divergent (UUT) and convergent (RAT) thinking scores were entered as dependent variables. Age, gender, and non-verbal general intelligence (RAPM) were entered as nuisance covariates. This analysis revealed a multivariate effect, reflecting an association between creativity and performance on weak associations [Pillai’s trace = 0.07,*F*(2, 107) = 4.03,*p*= .02] and semantic feature matching [Pillai’s trace = 0.066,*F*(2, 107) = 3.78,*p*= .026], as well as non-verbal intelligence [Pillai’s trace = 0.06,*F*(2, 107) = 3.42,*p*= .036]. These results established that tasks with high*semantic control*demands (weak association matching and feature selection), but not the strong association task with low semantic control demands, varied significantly with divergent and convergent creativity.

**Fig. 2.**
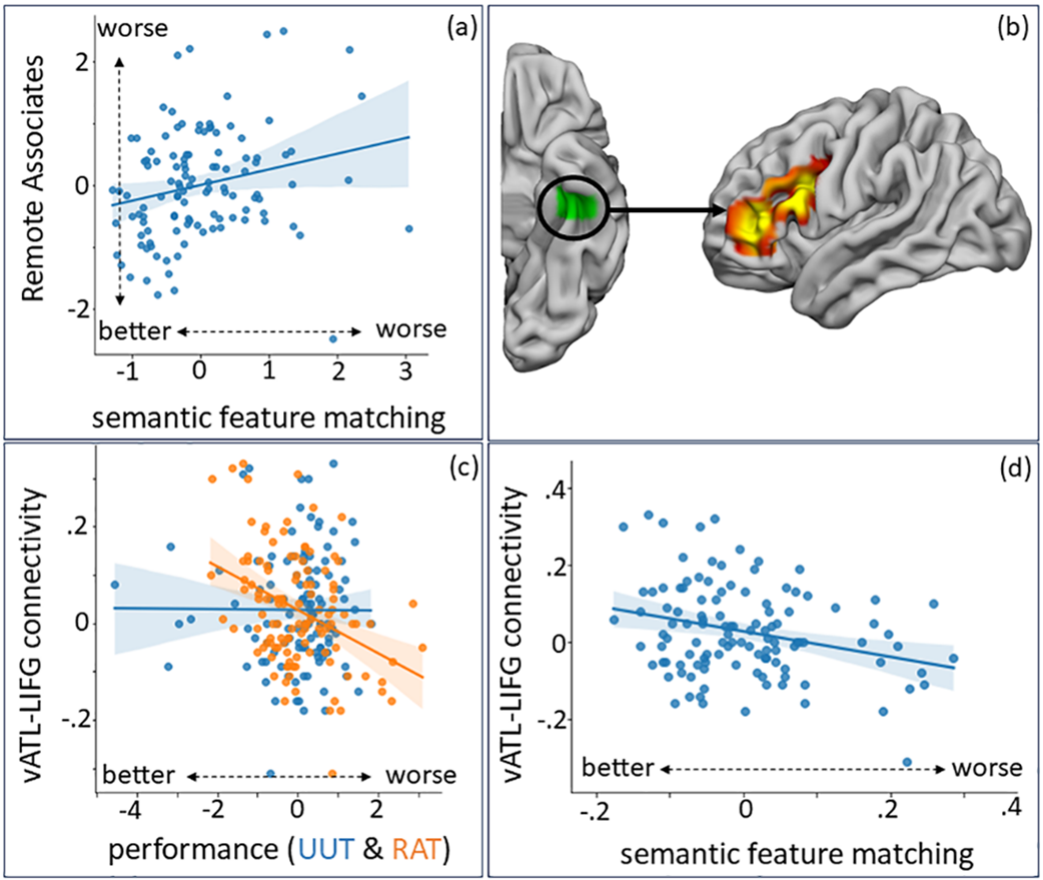
All values in the scatterplots are z-scored. (a) Partial regression plot of the significant positive relationship between performance on the remote associates task (RAT) and semantic feature matching; (b) connectivity from ATL (in green) to LIFG significantly associated with RAT performance and also plotted in c; (c) the significant relationship between RAT (but not UUT) performance and resting-state connectivity of the vATL (semantic representation) seed to the LIFG cluster; (d) semantic feature matching performance significantly correlates with coupling of the vATL to LIFG uncovered in the RAT seed-to-voxel analysis.

**Fig. 3.**
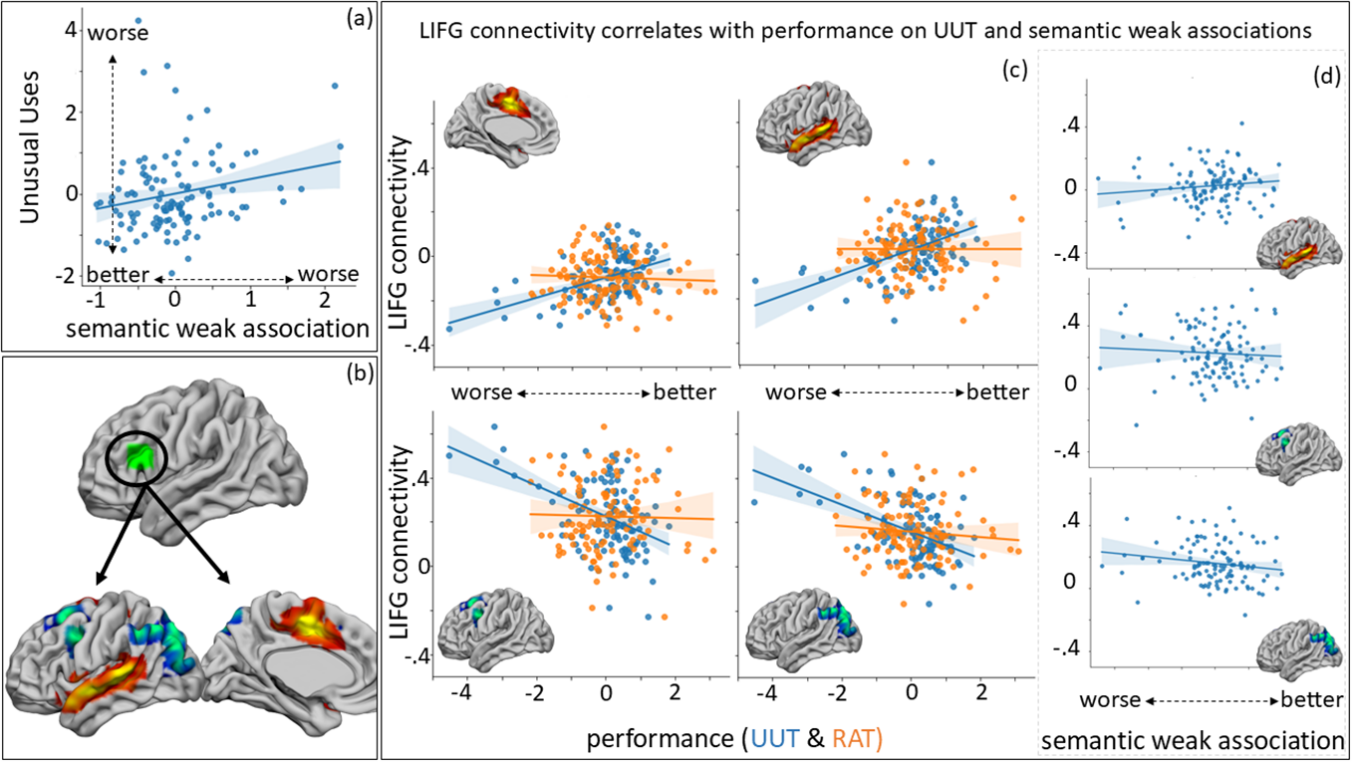
All values in the scatterplots are z-scored. (a) Partial regression plot of the significant relationship between performance on the unusual uses task (UUT) and semantic weak association; (b) the relationship between UUT performance and connectivity from LIFG (in green) to four clusters uncovered in the resting-state seed-to-voxel analysis and also plotted in c; (c) the significant relationship between UUT (but not RAT) performance and resting-state connectivity of the LIFG (semantic control) seed to each cluster identified in b; (d) weak association performance significantly correlates with connectivity from LIFG to LOC, SFG, and STG (uncovered in UUT seed-to-voxel analysis).

To better understand the relationship between aspects of semantic control and creativity, we examined the parameter estimates for these multivariate effects. Weak association correlated with performance on the unusual uses but not remote associates task (RAT:*b*= −6.669E-5, 95% CI = [0.000, 1.685E-5],*p*= .116; UUT:*b*= −3.436E-5, 95% CI = [-6.227E-5, -6.452E-6],*p*= .016). Feature matching showed the opposite pattern, correlating with remote associates, but not unusual uses, performance (RAT:*b*= −5.999E-5, 95% CI = [0.000, 1.159E-5],*p*= .016; UUT:*b*= 9.451E-6, 95% CI = [-6.722E-6, 2.562E-5],*p*= .249). These analyses revealed that the controlled retrieval of weak associations within semantic knowledge was associated with the divergent creativity task (Fig. 3), while goal-driven semantic selection was linked to convergent creativity (Fig. 2).

The behavioural results established that different aspects of semantic control were linked to distinct elements of creativity. The next stage of our analysis investigated whether individual differences in intrinsic connectivity with the neural semantic architecture were associated with performance on convergent and divergent creativity tasks. This analysis used seeds in ventral anterior temporal lobe (vATL), linked to storing multimodal conceptual representation, and left inferior frontal gyrus (LIFG), at the site of the strongest peak in a recent semantic control meta-analysis (Jackson, 2021). We used these seeds to identify areas of the brain functionally connected to key semantic control and representation areas and assessed where these connectivity patterns varied in relation to remote associates and unusual uses performance.

### 3.2 Seed-to-voxel connectivity analyses

We examined whether connectivity from two sites linked to semantic cognition (ventral anterior temporal lobe, vATL, and left inferior frontal gyrus, LIFG) correlated with performance on divergent and convergent creativity tasks. Better performance on the RAT was linked to greater coupling between vATL and a large cluster in left inferior frontal gyrus (LIFG), which sits within the semantic control network (Fig. 2b, c). While better performance on the unusual uses task was linked to greater coupling between the left inferior frontal gyrus (LIFG) and auditory-motor regions (i.e., SMA and STG), as well as the DMN and ventral attention network (VAN). It was also linked to greater de-coupling with frontoparietal (FPN) and default mode (DMN) network sites (i.e., SFG, LOC;Fig. 3b, c).

### 3.3 Connectivity results and semantic cognition

In a final step, we assessed whether the connectivity profiles uncovered by our analysis of creativity also correlated with semantic control. We extracted the connectivity values between vATL and the LIFG cluster, and LIFG and the clusters in pSTG, SMA, LOC, and SFG, and measured whether connectivity between these sites also correlated with performance on our semantic control tasks. We used a multivariate linear regression (SPSS; version 26), in which the connectivity values from vATL and LIFG to the clusters uncovered in the seed-to-voxel analysis (i.e., vATL-LIFG, LIFG-pSTG, LIFG-SMA, LIFG-LOC, LIFG-SFG) were dependent variables, and performance on semantic tasks (efficiency scores for feature matching, weak and strong association) was entered as explanatory variables. This analysis revealed a multivariate effect, reflecting an association between connectivity and performance on weak associations [Pillai’s trace = 0.109,*F*(5, 107) = 2.6,*p*= .029] and semantic feature matching [Pillai’s trace = 0.107,*F*(5, 107) = 2.6,*p*= .031], but not strong associations [Pillai’s trace = 0.042,*F*(5, 107) < 1]. These results established that tasks with high*semantic control*demands (weak association matching and feature selection), but not the strong association task, with low semantic control demands, varied significantly with the connectivity results uncovered in the resting-state creativity analysis.

To better understand the relationship between aspects of semantic control and our connectivity results, we examined the parameter estimates for these multivariate effects. Semantic feature matching correlated with connectivity from vATL-LIFG (*b*= -7.4E-5, 95% CI = [0, -2.63E-5],*p*= .003;Fig. 2d), as well as two sites uncovered from the unusual uses connectivity analysis (LIFG-LOC(UUT):*b*= −5.36E-5, 95% CI = [0, -1.83E-6],*p*= .043; LIFG-SFG(UUT):*b*= -6.52E-5, 95% CI = [0, 3.4E-7],*p*= .051;Supplementary Fig. S4). Weak association performance correlated with connectivity from LIFG to pSTG, LOC, and SFG, but not SMA, and did not correlate with vATL-LIFG connectivity (uncovered in the RAT analysis; vATL-LIFG(RAT):*b*= -1.69E-5, 95% CI = [-9.79E-5, 6.41E-5],*p*= .68; LIFG-SMA(UUT):*b*= −2.11E-6, 95% CI = [-7.02E-5, 7.44E-5],*p*= .95; LIFG-pSTG(UUT):*b*= 0, 95% CI = [0, -1.97E-5],*p*= .015; LIFG-LOC(UUT):*b*= 0, 95% CI = [3.6E-5, 0],*p*= .006; LIFG-SFG(UUT):*b*= 0, 95% CI = [6E-6, 0],*p*= .039;Fig. 3d). These analyses revealed that the controlled selection of relevant semantic features was associated with the connectivity patterns uncovered for RAT performance (Fig. 2d), as well as the connectivity of LIFG to SFG and LOC (Supplementary Fig. S4), linked to better divergent thinking. Retrieval of weak associations within semantic knowledge was associated with the connectivity patterns linked to better divergent creativity performance, but not convergent creativity (Fig. 3d).

## 4 Discussion

This study establishes specific relationships between different aspects of semantic control and verbal creativity, by showing that feature selection is associated with neurocognitive mechanisms underpinning convergent creativity (i.e., the ability to identify remote associates that link together words), while the controlled retrieval of weak associations relates to divergent creativity (i.e., generating unusual uses for objects). In addition to this behavioural dissociation, we demonstrate that the intrinsic neural architecture of sites implicated in semantic cognition relates to performance on these creativity tasks in distinctive ways. Increased connectivity between ventral anterior temporal lobe (vATL), associated with long-term storage of multimodal conceptual representations, and left inferior frontal gyrus (LIFG), a key semantic control site, is linked to better convergent creativity; while better divergent creativity is linked to increased coupling from LIFG to sensorimotor regions (superior temporal gyrus, STG, and supplementary motor area, SMA), as well as DMN and VAN, and reduced coupling to regions that largely overlap with the DMN and frontoparietal networks (lateral occipital cortex, LOC, and superior frontal gyrus, SFG).

While previous studies have linked aspects of semantic memory to convergent and divergent creativity, our novel contribution is to dissect the role of semantic control to verbal creativity specifically. We build on recent studies showing the importance of semantic cognition to creativity, drawing on contemporary models that distinguish between component semantic processes, including long-term conceptual knowledge and semantic control processes that support the controlled retrieval of weak aspects of knowledge and semantic selection. A recent study byOvando-Tellez, Kenett, et al. (2022)asked participants to list associations to polysemous words (e.g., bank—which could mean riverbank or financial institution), and used principal components analysis, to derive two variables which captured (i) the clustering of responses within semantic meanings and (ii) the ability to switch between meanings. They were able to relate these PCA components to performance on divergent and convergent creativity tasks. They found that the clustering of word meanings was associated with unusual uses performance (specifically, fluency and the number of unique ideas generated), and we add to this finding by showing that controlled access to associative semantic memory (i.e., not associative memory in general) relates to better divergent thinking. In line with this, we recently found that when participants generated unique associations between two words, activation in dorsomedial PFC, a key semantic control site, correlated with UUT performance (Krieger-Redwood et al., 2022). Our findings also demonstrate how the semantic intrinsic functional architecture of the brain relates to better divergent creativity and controlled semantic association. Together, these findings suggest that generating unusual uses for an object relies on semantic control processes that guide spreading activation of associations to a concept.

In contrast,Ovando-Tellez, Kenett, et al. (2022)found that the ability to switch between meanings of a polysemous word related to the convergent creativity processes required to combine remote associates to identify a linking word (Ovando-Tellez, Kenett, et al., 2022). Our findings similarly suggest that task-appropriate selection of specific semantic information supports convergent creativity. Taken together, the results ofOvando-Tellez, Kenett, et al. (2022)and ours suggest that the processes engaged for convergent creativity relate to the ability to manipulate the semantic system to direct activation away from association and towards different aspects of a meaning, whether it be an alternative definition or specific features of the concept. Furthermore, successful convergent problem solving has been linked to more flexible and inter-connected semantic information (with shorter paths and high connectivity between concepts;Luchini et al., 2023); the current study indicates this flexible structure is linked to strong semantic selection.Beaty and Kenett (2023)also suggest that creative participants possess the ability to use goal-directed leaps in semantic space, expanding the search space, while by-passing highly associated items. This seems particularly important for successful completion of both the RAT and the semantic feature selection task, where related items are irrelevant to the current goal (i.e., selection based on features, not association). This goal-directed search of the semantic space has previously been suggested to engage domain-general executive processes (Badre et al., 2005;Duncan & Owen, 2000;Fedorenko et al., 2013). However, our resting-state analysis suggests that successful goal-directed search and selection within the semantic system relies on semantic regions engaged for representation (vATL) and semantic control (LIFG).

The studies ofOvando-Tellez, Kenett, et al. (2022),Luchini et al. (2023), and our own highlight the multi-faceted nature of creativity, and elucidate the ways in which distinct aspects of semantic control relate to this capacity. Studies have also linked intrinsic connectivity in default mode (DMN), executive (e.g., frontoparietal; FCPN), ventral attention, and salience networks to successful creative ability (Beaty et al., 2014,^2015^,^2016^,^2019^,^2020^), but have not investigated the connectivity of sites specifically linked to semantic cognition. The vATL is central to semantic long-term memory (Balgova et al., 2022;Binney et al., 2016;Hoffman et al., 2015;Lambon Ralph et al., 2009,^2010^,^2015^,^2017^;Malone et al., 2016;Rice et al., 2015;Rice, Caswell, et al., 2018), and previous studies have shown that individual differences in the intrinsic connectivity of this site are associated with semantic performance (Gonzalez Alam et al., 2021;Mollo et al., 2016;Wu & Hoffman, 2024). Here, we demonstrate that better convergent creativity (i.e., strong remote associates performance) is linked to stronger coupling between vATL (associated with long-term semantic memory) and LIFG (associated with semantic control). This connectivity pattern is also associated with better performance on semantic feature matching, but not the retrieval of weak associations. These findings align with and extend a recent study which found that switching between different word meanings relates to DMN–executive coupling (Ovando-Tellez et al., 2023).

We found a different pattern of intrinsic functional connectivity was associated with divergent creativity on the unusual uses task. Participants who generated more unusual uses for common objects had stronger connectivity at rest between LIFG and sites associated with auditory-motor processing, DMN, and ventral attention networks (STG and SMA). These findings align with a recent task-based functional neuroimaging study that found a left-lateralised network, including LIFG, SMA, and STG, was activated as participants generated more original responses in bi-association tasks (Benedek et al., 2020). SMA has been associated with creativity across a range of tasks:Matheson and Kenett (2020)suggest that motor simulation supports the generation of ideas, which are then constrained by higher-level networks (such as the DMN and control networks) to arrive at the best response. This suggestion is broadly consistent with theories of semantic cognition, which suggest that sensory-motor features and abstract multimodal representations interact to guide context-appropriate behaviour (e.g.,Barsalou, 2008,^2016^;Fernandino & Binder, 2024;Lambon Ralph et al., 2017;Martin, 2016).

Better performance on the unusual uses task was also linked to*decoupling*of LIFG, a key region for semantic control, from regions of default mode, frontoparietal, attention and visual networks (within SFG, and LOC). The relationship between control and DMN networks has often been characterised as “antagonistic,” with one network activating while the other deactivates (Fox et al., 2005). While DMN is often implicated in internally focused memory, control networks are typically associated with externally directed attention. However, both creativity and semantic tasks require a combination of control and memory retrieval (Chiou et al., 2018;Davey et al., 2016;Wang et al., 2020), andBeaty et al. (2021)found that while executive and DMN regions were anti-correlated at rest, they reconfigured positively when participants were thinking creatively during a divergent thinking task; a similar pattern has also been observed for the retrieval of weak associations (Wang et al., 2024).Beaty and Kenett (2023)suggest that the interaction of these networks guides creative behaviour by supporting the generation of ideas (via associative thinking; DMN), identifying promising ideas (salience), and evaluating and selecting/modifying these ideas (executive). In our study, decoupling between LIFG and DMN/control also correlated with performance on the weak association task, mirroring the behavioural finding that UUT and weak associations share some common processes, while coupling of these networks correlated with better performance on the feature matching task. This suggests that it may be important for coupling of LIFG with DMN/control to change flexibly dependent on the patterns of retrieval needed to suit the current context (e.g., spreading activation for association, goal-directed selection for feature-based judgements). Furthermore, we did not find an anterior versus posterior LIFG distinction for association versus feature semantics, rather, our findings suggest that LIFG coupling may change to fit the context, given the finding that LIFG coupling with pSTG and SMA and decoupling to LOC and SFG correlated with better divergent creativity, and weak semantic association (except SMA), and LIFG and vATL coupling correlated with convergent creativity and feature semantics. This suggests that changing connectivity patterns between LIFG and representation, control, and DMN regions could be more important than a hard posterior/anterior distinction.

A recent study fromHerault et al. (2024)provides further evidence supporting the role of the SCN in divergent creativity. They found that judging distantly related semantic items activated the semantic control network (and DMN), while deactivating the multiple demand network (MDN); and this activation correlated with better alternative uses performance and creative behaviour. We also found that coupling between LIFG and DMN/control related to better performance on both the UUT and semantic association judgements, although in our study it was decoupling between the two. This decoupling might facilitate more unconstrained retrieval patterns to enable creative responses to surface and allow remotely related items to be associated. This might also explain why we did not find a relationship between vATL-LIFG connectivity and the unusual uses and semantic association tasks: this would allow the vATL to “broadcast” that is, allow multiple candidates to surface—a process necessary for the generation of unusual uses to an object, as well as relating remotely associated items. This idea finds support in a recent study which found that when participants generated associations for remotely related concepts, semantic control regions separated from the DMN to generate more flexible and original responses (Gao et al., 2022); a recent study fromChiou et al. (2018)demonstrated how connectivity between semantic control and representation (as well as “spoke” regions for feature selection) changed dependent on the type of decision made (i.e., association vs. feature).

There are of course some important limitations of this research. While we used classic assessments of convergent and divergent creativity, future research could use a wider range of creativity tasks and identify components of creativity in a more data-driven way. The inclusion of more diverse semantic assessments would similarly help to establish the characteristics of tasks that specifically link to convergent and divergent creativity. It is also important to note that, while both tasks may require some degree of inhibition (i.e., to reduce the influence of automatic spreading activation to the probe), the feature matching task also used an explicit lure to increase the inhibition demands, therefore, the tasks we used varied on more than one explicit dimension. Two recent studies indicate that even without the use of explicit lures, activation and connectivity differences between weak association and semantic feature selection still emerge (Chiou et al., 2018;Wang et al., 2024), suggesting that explicit distractors alone cannot account for the differences we found. Even so, future studies would benefit from reducing the number of control processes that change between task conditions in order to obtain a clear picture of whether it is, for example, semantic selection or inhibition that is the critical component for successful convergent creativity (e.g., RAT performance).

To further refine the relationship between convergent creativity and semantic control, future studies could also investigate whether different versions of the RAT might relate to different aspects of semantic control. This study used the compound RAT (i.e., participants generate compound words) and demonstrates that successful completion of the compound RAT shares the same semantic control processes, and underlying intrinsic architecture, required for successful semantic feature selection. The processes required for successful completion of the combination of associates task (CAT) might relate differently to semantic control than the compound RAT: an issue our study cannot address. For example, it is possible that the CAT might leverage the same semantic control processes (and functional neural architecture) as weak association, due to the associative nature of the CAT. However,Ovando-Tellez, Kenett, et al. (2022)used the CAT in their study and found a significant relationship with switching, not clustering of meaning, suggesting that even with use of the CAT, the relationship we uncovered with semantic feature selection might remain. This is an avenue that merits further exploration. In addition, intrinsic connectivity cannot establish how connectivity is dynamically altered to support aspects of semantic control and creativity. Future task-based fMRI research might show overlapping connectivity changes for divergent creativity and weak associations, and for convergent creativity and feature selection, in line with the dissociations we identified in both behavioural data and intrinsic connectivity.

In conclusion, this study extends our understanding of the relationship between semantic cognition and verbal creativity by demonstrating that separable aspects of semantic control relate to convergent and divergent creativity. Importantly, it also deepens our understanding of how the semantic intrinsic neural architecture of the brain relates to divergent and convergent creativity.

## Supplementary Material

Supplementary Material

## Data Availability

The connectivity maps were uploaded to Neurovault (https://neurovault.org/collections/RUIVEQNY/;Gorgolewski et al., 2015). The conditions of our ethics approval do not permit public archiving of the raw MRI data supporting this study. Readers seeking access to these data should contact the lead author, Katya Krieger-Redwood, the PI Professor Beth Jefferies, or the local ethics committee at the Department of Psychology and York Neuroimaging Centre, University of York. Access will be granted to named individuals in accordance with ethical procedures governing the reuse of sensitive data. Specifically, the following conditions must be met to obtain access to the data: approval by the Department of Psychology and York Neuroimaging Research Ethics Committees and a suitable legal basis for the release of the data under GDPR. Scripts used for running the tasks used in this study are uploaded to OSF (https://osf.io/dy37q/?view_only=114b10e2fedc426f841cc615f7e6931d).
