# Supplementary material for: Divergent and convergent creativity relate to different aspects of semantic control
Source: Imaging Neurosci (Camb). 2025 Mar 7;3:imag_a_00502. doi: 10.1162/imag_a_00502 (PMC12320018; doi:10.1162/imag_a_00502)
Supplement: Supplementary Material [file imag_a_00502-supp.pdf]

Supplementary Materials

Psycholinguistic properties of the stimuli:

| | Strength of association | | | |
|---|---|---|---|---|
| | Strong | Weak | t | Sig. |
| | Mean (standard error) | | | |
| **Word Length** | 6.43 (.39) | 6.6 (.34) | -.16 | .873 |
| **Lexical Frequency** | 13564.8 (1887) | 11233.6 (1805) | .89 | .374 |
| **Familiarity** | 6.02 (.09) | 6.12 (.08) | -.88 | .381 |
| **Imageability** | 5.16 (.13) | 4.96 (.13) | 1.07 | .287 |
| **Semantic Association** | 6.02 (.07) | 3.32 (.10) | 21.74 | .000 |

Table S1. Psycholinguistic variables for our semantic battery by strength of association.

| | Task | | | |
|---|---|---|---|---|
| | Feature Match | Weak Association | t | Sig. |
| | Mean (standard error) | | | |
| **Word Length** | 6 (.27) | 6.6 (.34) | -1.45 | .149 |
| **Lexical Frequency** | 18696.4 (1214.3) | 11233.6 (1805) | .61 | .544 |

Table S2. Psycholinguistic variables for our feature matching battery (with weak semantic association (i.e., our other aspect of semantic control) for comparison).

Remote Associates Task Instructions:

Instructions to participant:

'In this experiment you will be presented with 30 words configurations consisting of 3 words each. After each presentation your task is to type one word you think all 3 presented words have in common. For example, an answer for the words [Foul, Ground, Mate] is [Play] (foul play, playground, playmate).\You will also be asked whether you have used insight in finding your answer, i.e. whether

you had a EUREKA! moment and just knew the answer immediately or whether you had to stop and think.\Please press the SPACEBAR to continue'

Semantic Task Behavioural Results:

Efficiency on all three semantic tasks, strong association is the most efficient followed by weak association and feature matching.

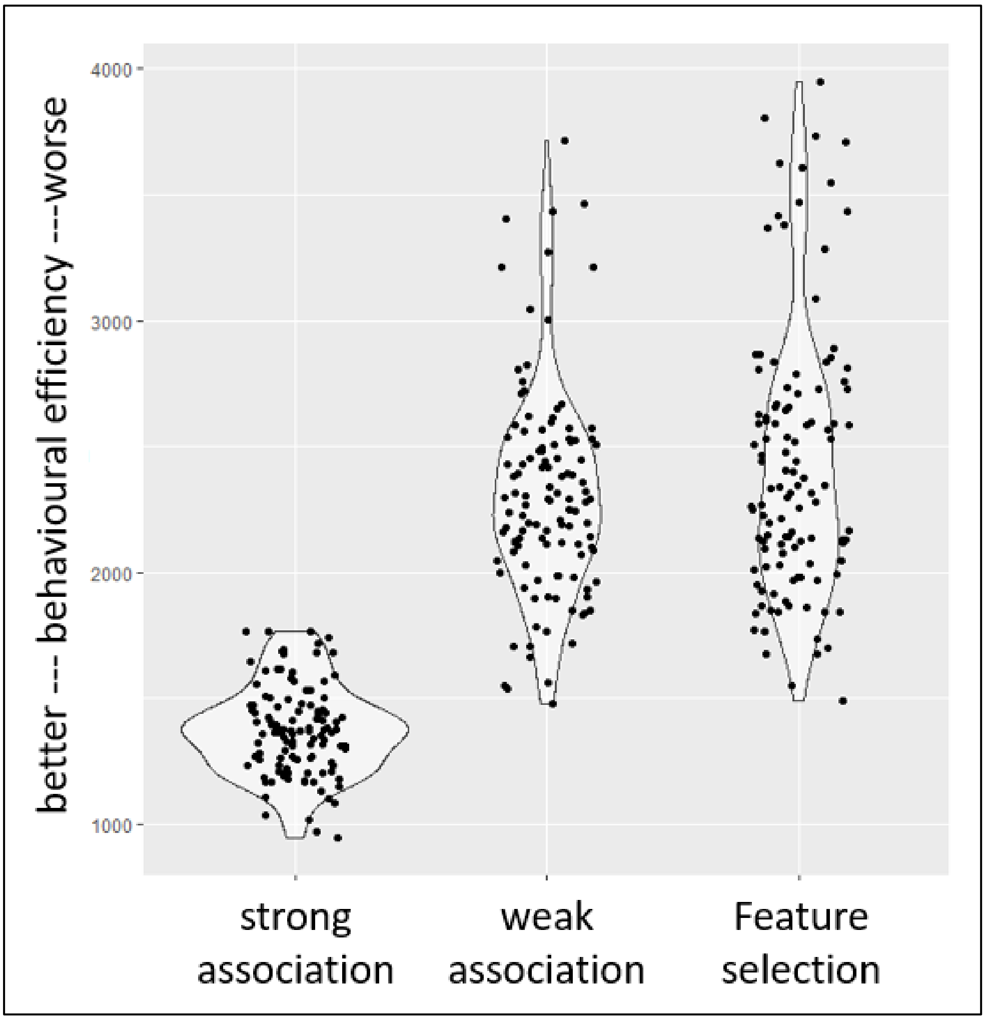


Figure S1. Behavioural efficiency scores for strong association, weak association, feature matching.

Correlations of UUT scoring methods:

| | Number of Unique Uses | Total Number of Responses | Average word2vec Score |
|---|---|---|---|
| Number of Unique Uses | 1 | | |
| Total Number of Responses | .266** | 1 | |
| Average word2vec Score | .540*** | 0.133 | 1 |

Table S3. Correlation is significant at the ** 0.01; ***.001 level (2-tailed).

Confirmation of RS-RAT result using binary 1/0 accuracy measure:

Our resting-state analysis found that connectivity between vATL and LIFG was linked to better performance on the remote associates task (RAT). We confirmed that the correlation between connectivity and RAT performance using the binary scoring method still stands: Pearson $r(115) = .37$, $p < .001$.

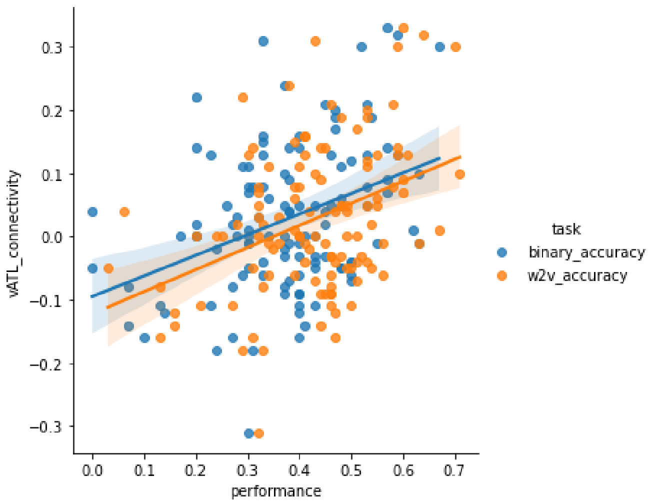


Figure S2. RAT accuracy measures plotted against vATL-LIFG connectivity.

## Our RAT Data Compared to RAT Norms:

| Our Participants | | | | | | | Norms | | | | | | | | | |
|---|---|---|---|---|---|---|---|---|---|---|---|---|---|---|---|---|
| RAT Items | | | | | | | 2sec (n=89) | 7 sec (n=85) | | | 15 sec (n=76) | | | 30 sec (n=39) | | |
| probe1 | probe2 | probe3 | solution | difficulty | word2vec | % | % | % | RT | SD | % | RT | SD | % | RT | SD |
| age | mile | sand | stone | hard | 30 | 30 | 11 | 27 | 5.02 | 1.25 | 32 | 8.35 | 2.83 | 44 | 16.61 | 8.67 |
| aid | rubber | wagon | band | easy | 50 | 50 | 22 | 56 | 4.21 | 1.5 | 75 | 5.41 | 2.15 | 69 | 6.51 | 4.62 |
| catcher | food | hot | dog | hard | 20 | 10 | 3 | 14 | 4.62 | 1.39 | 30 | 8.04 | 3.26 | 46 | 10.22 | 5.54 |
| chamber | mask | natural | gas | hard | 30 | 20 | 7 | 26 | 3.93 | 1.13 | 33 | 5.86 | 2.25 | 44 | 5.27 | 4.9 |
| cottage | swiss | cake | cheese | easy | 70 | 60 | 52 | 84 | 3.28 | 1.25 | 96 | 3.96 | 2.37 | 064* | 10.85 | 7.04 |
| cracker | fly | fighter | fire | easy | 70 | 70 | 17 | 45 | 4.95 | 1.54 | 68 | 5.91 | 2.54 | 85 | 6.12 | 3.87 |
| cross | rain | tie | bow | hard | 40 | 40 | 3 | 18 | 5.54 | 1.24 | 34 | 8.56 | 3.25 | 46 | 13.75 | 8.39 |
| dew | comb | bee | honey | easy | 70 | 70 | 30 | 66 | 4.24 | 1.5 | 80 | 5.63 | 3.15 | 1000 | 4.12 | 2.14 |
| dream | break | light | day | easy | 60 | 60 | 24 | 56 | 4.36 | 1.49 | 64 | 5.35 | 2.56 | 56 | 7.91 | 6.72 |
| dress | dial | flower | sun | medium | 30 | 30 | 4 | 15 | 4.45 | 1.62 | 29 | 5.79 | 2.74 | 51 | 7.78 | 5.72 |
| fish | mine | rush | gold | easy | 40 | 40 | 17 | 46 | 4.27 | 1.09 | 63 | 6.48 | 3.32 | 74 | 9.07 | 6.83 |
| flake | mobile | cone | snow | easy | 20 | 10 | 9 | 47 | 4.2 | 1.61 | 71 | 6.72 | 3.1 | 79 | 8.68 | 7.02 |
| flower | friend | scout | girl | medium | 30 | 20 | 9 | 22 | 5.26 | 1.47 | 51 | 8.06 | 2.94 | 67 | 11.43 | 7.7 |
| force | line | mail | air | hard | 10 | 10 | 10 | 27 | 3.94 | 1.25 | 28 | 7.52 | 4.06 | 28 | 13.9 | 7.76 |
| foul | ground | mate | play | hard | 90 | 80 | 2 | 6 | 4.81 | 1.26 | 25 | 8.12 | 2.87 | 46 | 9.33 | 6.85 |
| french | car | shoe | horn | medium | 30 | 30 | 9 | 29 | 4.9 | 1.49 | 34 | 6.88 | 2.57 | 69 | 12.58 | 8.71 |
| light | birthday | stick | candle | medium | 50 | 50 | 8 | 36 | 5.14 | 1.65 | 41 | 7.97 | 3.02 | 46 | 9.74 | 6.83 |
| loser | throat | spot | sore | easy | 60 | 60 | 22 | 61 | 4.19 | 1.49 | 86 | 5.38 | 3.02 | 82 | 6.31 | 4.06 |
| main | sweeper | light | street | medium | 30 | 20 | 12 | 32 | 4.7 | 1.35 | 33 | 5.73 | 2.82 | 64 | 7.7 | 5.65 |
| measure | worm | video | tape | easy | 70 | 60 | 10 | 45 | 4.74 | 1.59 | 58 | 6.12 | 2.9 | 87 | 8.36 | 5.24 |
| office | mail | hat | box | hard | 20 | 10 | 2 | 14 | 6.03 | 0.83 | 32 | 8.26 | 3.74 | 21 | 17.23 | 7.6 |
| opera | hand | dish | soap | medium | 20 | 10 | 16 | 33 | 5.26 | 1.41 | 47 | 6.54 | 3.11 | 62 | 7.92 | 6.45 |
| pie | luck | belly | pot | medium | 40 | 40 | 15 | 38 | 4.35 | 0.93 | 49 | 5.31 | 2.03 | 44 | 8.68 | 4.36 |
| right | cat | carbon | copy | hard | 10 | 10 | 6 | 25 | 4.84 | 1.78 | 39 | 7.45 | 3.14 | 46 | 11.88 | 7.43 |
| rocking | wheel | high | chair | easy | 60 | 60 | 37 | 73 | 3.98 | 1.43 | 80 | 4.98 | 2.55 | 87 | 5.84 | 5.36 |
| sage | paint | hair | brush | medium | 30 | 30 | 8 | 28 | 5.3 | 1.35 | 34 | 7.04 | 2.72 | 69 | 9.88 | 6.87 |
| sense | courtesy | place | common | medium | 50 | 50 | 8 | 33 | 4.67 | 1.29 | 54 | 6.28 | 2.89 | 67 | 9.24 | 8.11 |
| shine | beam | struck | moon | medium | 30 | 10 | 3 | 22 | 4.55 | 1.8 | 41 | 6.08 | 2.62 | 62 | 6.17 | 4.93 |
| stick | maker | point | match | hard | 20 | 20 | 1 | 4 | 5.42 | 1.23 | 46 | 6.65 | 3.46 | 21 | 12.19 | 8.15 |
| tank | hill | secret | top | hard | 20 | 10 | 2 | 15 | 5.81 | 0.59 | 30 | 10.13 | 3.1 | 38 | 11.2 | 5.82 |

note: w2v has been multiplied by 100 to match the other metrics of ACC

Table S4: Trial data from our study, alongside normative RAT data (Bowden & Jung-Beeman, 2003). Triads in our study were presented on screen for 5 seconds and participants were required to type their answer following an instruction to do so.

## Partial correlations controlling for: age, gender, RAPM, strong association:

| | weak association | feature matching | RAT | UUT |
|---|---|---|---|---|
| weak associations | 1 | | | |
| feature matching | 0.281** | 1 | | |
| RAT | -0.217* | -0.276** | 1 | |
| UUT | -0.206* | 0.046 | 0.06 | 1 |

Table S5.

**$p$ < .005, * $p$ < .05

**Overlap of thresholded maps with existing networks**.

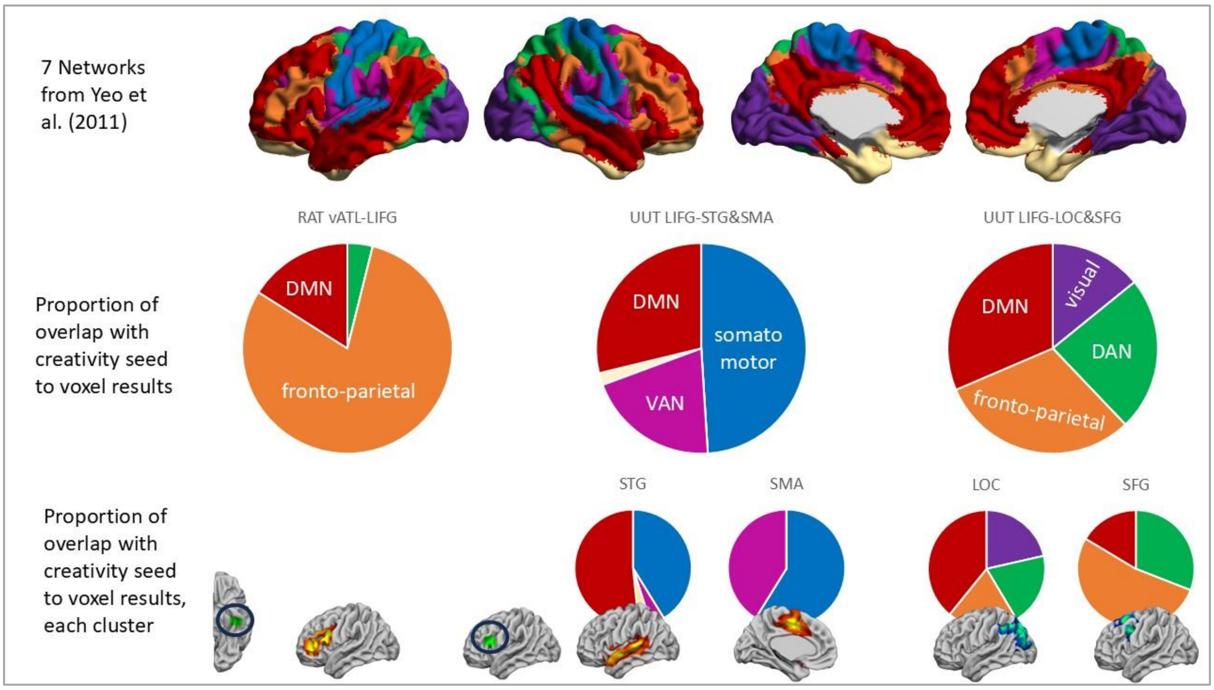


Figure S3. Overlap of seed-to-voxel analyses with the resting state networks derived by Yeo et al. (2011). Seeds are in green, with a circle around them.

Overlap with SCN

| Cluster (seed) | % overlap with SCN |
|---|---|
| LIFG (vATL) | 68% |
| LOC (LIFG) | 0% |
| SFG (LIFG) | 10% |
| SMA (LIFG) | 0% |
| STG (LIFG) | 8% |

Table S6. Percentage overlap of each cluster resulting from seed-voxel analyses and the semantic control network (SCN).

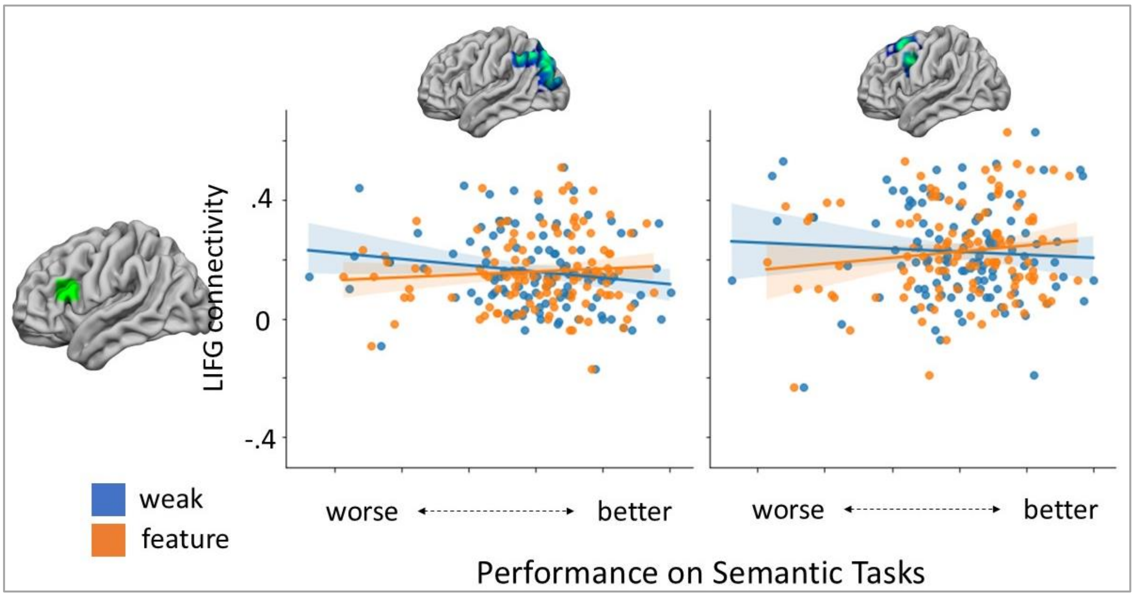


Figure S4. Feature matching and weak association performance significantly correlates with connectivity from LIFG to LOC and SFG (uncovered in UUT seed-to-voxel analysis).

**Battery of Tasks**

The tasks analysed in this study were run alongside a battery of other tasks, which are listed here.

Semantic tasks: Relatedness Task (Word-to-Word Matching; Picture-to-Picture Matching), Identity Matching Task (Word-to-Picture Matching), Scrambled Picture Matching.

Non-semantic tasks: Word Pair Memory Task (WPMT; measure of episodic memory); Verbal Fluency Task (Letter and Category Fluency); Flanker Task; Task Switching Task; Four Mountains Task; Digit Span.

<u>Additional Connectivity Analyses</u>

For completeness, we performed an additional connectivity analysis, where we included three ROI's: (i) pMTG, the second strongest site implicated in semantic control, from the Jackson et al. 2021 meta-analysis of semantic control; and two LIFG sites from Badre et al. (2005), implicated in (ii) controlled retrieval and (iii) controlled selection of semantic information. We used two-sided tests to determine significant clusters. Group-level analyses in CONN were cluster-level FWE corrected and controlled for the number of seeds (Bonferroni, $p < .017$), and used a height threshold of $t > 2.6$).

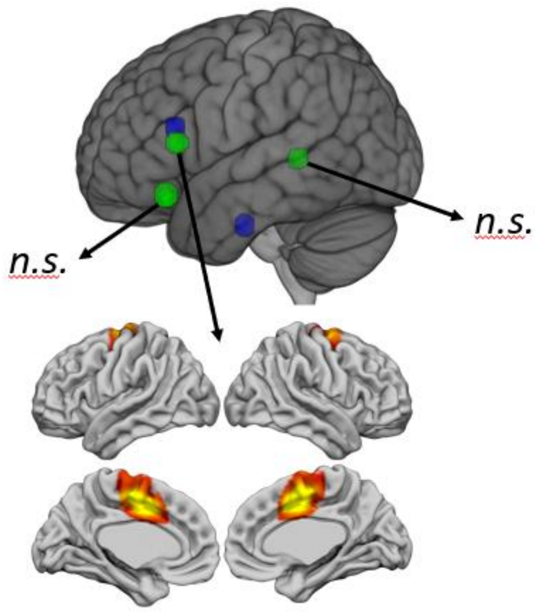


Figure S5. The ROI's in our main analysis are included in blue, for reference. The ROI's included in this analysis are in green. We placed 6mm spheres around: the LIFG sites from Badre et al.'s (2005) peak for selection (posterior LIFG; -54, 21, 12) and retrieval (anterior LIFG; -45, 27, -15); as well as the pMTG peak from Jackson et al.'s (2021) meta-analysis of semantic control (-54, -42, 4).
